# Associations of negative cognitions, emotional regulation, and depression symptoms across four continents: International support for the cognitive model of depression

**DOI:** 10.1186/s12888-019-2423-x

**Published:** 2020-01-13

**Authors:** Saghar Chahar Mahali, Shadi Beshai, Justin R. Feeney, Sandeep Mishra

**Affiliations:** 1 Department of Psychology, University of Regina, 3737 Wascana Parkway, Regina, Saskatchewan S4S0A2 Canada; 2 Faculty of Business Administration, University of Regina, Regina, Canada; 3 Department of Management, Lang School of Business and Economics, University of Guelph, 50 Stone Road East, Guelph, Ontario N1G 2W1 Canada

**Keywords:** Depression, Negative automatic thoughts, Dysfunctional attitudes, Culture, Alignment method, Emotion regulation, Suppression, Cognitive reappraisal

## Abstract

**Background:**

Cognitive-behavioral therapy (CBT) is one of the most widely tested and empirically supported psychological treatments for depression. Beck and other scholars established the theoretical foundations of CBT among North American populations, yet surprisingly few studies have examined central hypotheses of the cognitive model of depression among people living in non-Western regions.

**Methods:**

In the present study, we used the alignment method to minimize measurement bias to examine several central hypotheses of the cognitive model among adults living on four continents (*n* = 752): North America (*n* = 103; female = 29.1%), Europe (*n* = 404; female = 36.4%), South America (*n* = 108; female = 18.5%), and Asia (*n* = 136; female = 19.9%).

**Results:**

Depressive symptoms were positively and strongly correlated with negative automatic thoughts about self (ATQ-N), and moderately associated with dysfunctional attitudes (DAS) among people living on the four continents. Further, use of emotional suppression strategies to regulate emotion (ERQ-Suppression) was moderately and positively associated with depressive symptoms among people on all four continents, while use of cognitive-reappraisal (ERQ-Reappraisal) was not systematically associated with depressive symptoms.

**Conclusions:**

Results of this study offer preliminary cross-continental support for foundational hypotheses of the cognitive model of depression. Negative thoughts appear to be associated with depression in all regions of the world, cementing this construct as a hallmark feature of the disorder.

## Background

Depression is a highly prevalent and disabling mental health condition [1]. Latest estimates from the World Health Organization place depression as the world’s leading cause of disability [2]. Fortunately, there are many efficacious treatments for depression [3], including cognitive-behavioral therapy [4, 5]. The theoretical underpinnings of treatments such as CBT have seldom been examined among people living in non-Western nations, or among people of non-European decent [6, 7]. Accordingly, the present study examined the validity of central hypotheses related to the cognitive model of depression [8] among people living on four continents: Asia, Europe, North America, and South America. In the following, we (a) briefly review the literature on the cognitive model of depression, the centrality of cognitions to cognitive therapy for the disorder, and on emotion regulation strategies and their use and consequences across cultures, and (b) present an empirical study examining whether predictions of the cognitive model hold cross-continentally.

Depression is a multifaceted condition, typified by behavioral, affective, cognitive, and somatic symptoms. According to the fifth edition of the Diagnostic and Statistical Manual (DSM-5, [9]), major depression is a condition wherein symptoms – low-mood, lack of pleasure in otherwise enjoyable activities, disruptions in sleep and appetite, low energy, poor concentration, suicidal thoughts, and feelings of worthlessness and guilt, etc. – have persisted for two or more weeks and have caused significant distress or impairment. Depression is a heterogenous disorder, and several combinations of these symptoms could be placed under the same diagnostic umbrella of depression.

Negative self-referent cognitions have long been theorized to be central to the experience of depression [8]. Beck’s seminal cognitive theory was first to formalize hypotheses regarding the role of cognitions in depression. Beck posited that there are several cognitive layers implicated in the onset and maintenance of depression [4, 10, 11]. Importantly, Beck hypothesized that, at the most accessible or proximate level of the information processing system, negative automatic thoughts about self (“e.g., “I am no good”; “I am unlovable”) are predominant in depression and are central to the experience of the condition [8]. This hypothesis is also known as cognitive triad hypothesis. Strong empirical evidence supports this claim among Western participants: negative self-referent thoughts have been identified as a consistent and defining feature of the disorder [12–14].

Many early studies conducted in the West with depressed or dysphoric (i.e., people showing elevated symptoms of depression) participants confirm the validity of the cognitive triad hypothesis during bouts of depression (reviewed in Clark & Beck [8]). However, there have been surprisingly few examinations of the cognitive triad hypothesis outside of Western regions, although preliminary evidence collected among single non-Western cultures is suggestive (reviewed in Beshai et al. 2012 [6]). Sami and El-Gawad [15] found that depressed Arab participants experienced relatively little guilt or self-deprecating cognitions. Similarly, [16] found that Pakistanis were less likely to present with guilt as a feature of their depression compared with their Austrian counters. In fact, Stompe et al. [16] specifically suggested that depressive self-reproach and negativity may only be a defining feature of depression among Judeo-Christian sufferers. Despite these inconsistent findings across cultures, negative automatic thoughts have been found to associate with depression symptoms among Chinese [17, 18] Egyptian [6, 7], Turkish [19], and Japanese [20] participants. However, none of these studies specifically compared and contrasted cognitive inputs into depression across multiple regions.

Beck’s model also posited more remote cognitive layers that are believed to be implicated in the maintenance of depression. For example, Beck and others hypothesized that people suffering from depression would hold dysfunctional attitudes, or “rules for living”, that are often rigid and negatively skewed (e.g., “If I don’t do everything perfectly, it means I am no good at all”). According to Beck’s cognitive model, people who are vulnerable to depression detect violation of these rules or attitudes (e.g., detect criticism from others), which results in activation of more proximate negative automatic thoughts, finally resulting in an amplified depressive experience. Weissman and Beck [21] created the Dysfunctional Attitude Scale (DAS) to assess dysfunctional attitudes characteristic of depressive thinking, and in turn, measure the level of activation and access to negative cognitive structures.

Substantial evidence suggests that dysfunctional attitudes lead to depressive symptoms among Western populations of European descent, as well as in non-Western populations. For example, Beshai et al. [7, 22] found that European-Canadian people with elevated depression symptoms reported higher dysfunctional attitudes than those with minimal symptoms. Other researchers have reported similar findings [23, 24]. The association of dysfunctional attitudes with depression symptoms has also been demonstrated across several countries around the world, such as Egypt [7], Malaysia [25], Romania [26], Spain [27], Iran [28], and United Arab Emirates [29]. Although these results are suggestive of a generalizable association between dysfunction attitudes and depressive symptoms, many of these studies have employed small, relatively underpowered samples, or were not comparative in their approach (i.e., no control).

Another foundational hypothesis of cognitive models of depression is that desired emotional and behavioral changes result from cognitive modification [30]. Accordingly, the skill of cognitive reappraisal – the reinterpretation of emotionally eliciting materials or events in a way that alters the emotional response [31] – is considered to be an adaptive and effective strategy to manage negative emotions. For example, in using reappraisal to regulate sadness in response to losing one’s job, someone might start to reinterpret the event of losing their employment as an opportunity to travel. By contrast, the regulation strategy of emotional suppression – defined as a person’s attempt to decrease emotional expression of a certain emotion and/or their attempt to decrease or eliminate thoughts or outward expression of this said emotion [32] – is considered to be maladaptive and ineffective in regulating negative emotions. For example, an individual who experiences sadness in reaction to losing their job might suppress this sadness by attempting to hide any discernible signs (i.e., facial expressions) of their sadness to their family and friends, or by externally expressing an opposite emotional reaction (e.g., “putting on a smile”).

Recent research among participants of European descent shows that cognitive reappraisal and emotional suppression have dramatically divergent affective, social, and cognitive consequences [33]. Suppression does not effectively lead to reduced negative affect, but paradoxically may increase sympathetic nervous system response. Cognitive reappraisal, by contrast, has the opposite effect in that it leads to decreases in negative emotions and decreased systemic activity [34]. Further, it appears that cognitive reappraisal is associated with decreased anxiety and depression symptoms, while suppression is associated with increases in symptoms of depression and anxiety [35–37]. However, the divergent effects of suppression versus cognitive appraisal have not been extensively investigated in diverse regional contexts.

Several studies suggest that culture moderates the emotional outcomes of emotion regulation. Soto, Perez, Kim, Lee and Minnick [38] found that adverse effects of suppression of emotion were observed among European students but not Asian students, suggesting that the maladaptiveness of this approach is culture specific. Similarly, Butler, Lee and Gross [39] found that, for participants with Western values (e.g., independence of self and individualism), suppression was associated with increase in negative emotion. However, this adverse emotional effect of suppression was not found for participants holding Asian values (e.g., interdependence of self and collective values). These findings were replicated by Kwon, Yoon, Joormann, and Kwon [40] among a sample of Korean participants. Taken together, the evidence suggests that identification of cultural heritage and level of assimilation into mainstream culture (and disengagement from one’s heritage culture) both moderate the adaptiveness different emotion regulation strategies.

### Overview

The evidence reviewed above suggests that (a) there are core cognitive processes that are central to depressive symptoms (i.e., negative self-referent thoughts; dysfunctional attitudes); (b) cognitive modification leads to amelioration of depressive symptoms; and (c) different cognitive modification strategies have divergent effects across cultures. In the present study, we examined the relationships of depression symptoms, negative automatic thoughts, dysfunctional attitudes, and emotional suppression and cognitive reappraisal in a large cross-continental sample of participants living on four continents and in several countries (Table 1). We used a state-of-the-art analysis to minimize measurement bias in examining such relationships. Specifically, we used the alignment method [41] to reduce invariance of the measures used across the four continents. This research replicates and extends extant literature in several important ways. First, this study is the first large scale cross-continental examination of central hypotheses of the cognitive model of depression. The cognitive model is the foundation upon which CBT and other cognitive therapeutic approaches rest, and previous cross-national investigations have examined either single populations (e.g., [29]) or pairs of populations (e.g., [6]). Unfortunately, the majority of depression interventions and their theoretical foundations have almost exclusively been developed by or with individuals of the majority culture (White; middle class; Judeo-Christian). Second, this is the first study to employ the alignment method for optimal scale performance across several continents. Potential measurement bias is an inherent problem in any study, but is particularly salient in cross-national investigations given diversity in interpretation of scale items. Finally, our sample size was large, and therefore, sufficiently statistically powered to test even small effects.

**Table 1:** Summary of countries

Continent (Total number of countries)	Country	n (%)
North America (3)	Canada	16 (15.5)
	United States	76 (73.8)
	Mexico	11 (10.7)
Europe (29)	Bosnia and Herzegovina	66 (16.3)
	Spain	20 (5.0)
	Italy	22 (5.4)
	Russia	44 (10.9)
	Serbia	82 (20.3)
	Ukraine	23 (5.7)
	Other	147 (36.4)
South America (8)	Brazil	24 (22.2)
	Argentina	6 (5.6)
	Venezuela	67 (62.0)
	Other	11 (10.2)
Asia (14)	India	58 (42.6)
	Turkey	29 (21.3)
	Vietnam	13 (9.6)
	Other	36 (26.5)

In accordance with the cognitive model of depression, we predicted that depression symptoms would be significantly and positively correlated with negative automatic thoughts and dysfunctional attitudes among people across cultures and continents. Further, we predicted that, among people living in North America and Europe, use of cognitive reappraisal strategies would be significantly and negatively associated with depression symptoms, and use of suppression as a strategy for emotion regulation would be positively associated with depression symptoms. This is a first step to understand the assumptions underlying CBT and its cross-continental adaptation.

## Method

### Procedure

Participants were recruited via the online crowdsourcing platform CrowdFlower. CrowdFlower and other similar platforms (e.g., Amazon’s Mechanical Turk) are online labour markets that have been used widely for behavioural and clinical research [42, 43]. Importantly, compared to Mechanical Turk, CrowdFlower has a more international reach given that the service utilizes multiple different source “channels” for workers (i.e., people who complete short tasks for compensation). Requesters can post computerized tasks which are disseminated through such channels. Workers can choose among available study tasks and select those that are appealing to them. Crowdsourced participants are more representative of the general population than other convenience-based sampling methods [44]. Compared to general population, crowdsourced participants tend to be more representative of Internet users, and therefore, “are typically younger, more educated, less religious, and more liberal” [44]. Mechanical Turk utilizes only its own platform, and so the vast majority of workers are based in only a handful of countries. Until recently, Mechanical Turk was only available to individuals living in the United States. Thus far, over 5 million people have completed over 1million tasks on CrowdFlower [45].

### Participants

Participants for this study were recruited from various countries across North America (*N* = 103) Europe (*N* = 404), South America (*N* = 108), and Asia (*N* = 136). There was no restriction based on country or region of origin for participating in this study. The initial sample consisted of 858 participants. One hundred and three participants were excluded from the study due to missing total scores on the four key scales (summarized below) used in this study, for not being from one of the four continents targeted in the study (noted above), or for not passing an included attention check question. The attention check question was worded as follows: “If you are reading this, please do not pick “Psychology”, and pick “Mindfulness” instead from the word choices provided”. Further, three participants were also excluded from the study for not meeting the inclusion criteria of being an adult (≥18 years of age). The final sample consisted of 752 participants (*M* = 31.52, *SD* = 9.07, *Range* 18–69). A total of 224 (29.8%) women participated in the study. For their participation, all participants received financial compensation ($1.50 USD) that was commensurate with compensation in other crowdsourcing studies [42]. The University of Regina Research Ethics Board (File# 2015–174, Date: 17/11/2015) approved this study prior to data collection. All participants provided written consent prior to completing the study, and the measures were all administered in English. The data collection took place in December 2015.

### Measures

#### Demographic information

All participants provided their age, gender, ethnicity, country of residence, marital status, annual income, religious affiliation, education level, and primary language spoken at home. A summary of pertinent demographics can be found in Table 2.

**Table 2:** Summary of demographics

	Entire Sample *n* = 752	North America *n* = 103	Europe *n* = 404	South America *n* = 108	Asia *n* = 136
Age: *M* (*SD*)	31.52 (9.07)	32.35 (10.16)	32.89 (8.80)	28.50 (9.47)	29.33 (7.56)
Sex: n (%)					
Female	224 (29.7)	30 (29.1)	147 (36.4)	20 (18.5)	27 (19.9)
Male	527 (70.1)	72 (69.9)	257 (63.6)	88 (81.5)	109 (80.1)
Other	1 (.1)	1 (1.0)			
Ethnicity					
White	575 (76.5)	80 (77.7)	395 (97.8)	70 (64.8)	30 (22.1)
Asian	113 (15.0)	11 (10.7)	5 (1.2)	1 (.9)	97 (71.3)
Other	64 (8.6)	12 (11.6)	4 (1.0)	37 (34.3)	9 (6.6)
Marital Status					
Single/ Never Married	416 (55.3)	53 (51.5)	214 (53.0)	73 (67.6)	75 (55.1)
Married	293 (39.0)	40 (38.8)	167 (41.3)	30 (27.8)	56 (41.2)
Separated/ Divorced	30 (4.0)	9 (8.7)	15 (3.7)	5 (4.6)	2 (1.5)
Other	13 (1.7)	1 (1.0)	8 (2.0)	1 (.9)	3 (2.2)
Education					
Secondary School or Equivalent	153 (20.3)	17 (16.5)	92 (22.8)	31 (28.7)	13 (9.6)
Below Bachelor	84 (11.2)	14 (13.6)	40 (9.9)	19 (17.6)	11 (8.1)
Bachelor’s Degree	201 (26.6)	29 (28.2)	92 (22.8)	18 (16.7)	62 (45.6)
Above Bachelor	91 (12.1)	13 (12.6)	45 (11.1)	18 (16.7)	14 (10.3)
Master’s Degree	131 (17.4)	16 (15.5)	76 (18.8)	9 (8.3)	30 (22.1)
Other	92 (12.1)	14 (13.6)	59 (14.6)	13 (12.0)	6 (4.3)
Annual Income					
Unemployed/ No Annual Income	163 (21.7)	7 (6.8)	107 (26.5)	26 (24.1)	23 (16.9)
$10,000 - $30,000	272 (36.2)	29 (28.2)	165 (40.8)	34 (31.5)	43 (31.6)
$30,000 - $50,000	108 (14.4)	24 (23.3)	43 (10.6)	16 (14.8)	25 (18.4)
$50,000 - $75,000	70 (9.3)	25 (24.3)	20 (5.0)	8 (7.4)	17 (12.5)
$75,000 and over	42 (5.6)	14 (13.6)	15 (3.7)	7 (6.5)	6 (4.4)
Other	97 (12.9)	4 (3.8)	54 (13.4)	17 (15.8)	22 (16.2)
Religion					
Christianity	417 (55.5)	70 (68.0)	258 (63.9)	72 (66.7)	16 (11.8)
Islam	92 (12.2)	2 (1.9)	41 (10.1)	NA	49 (36.0)
Atheism	91 (12.1)	10 (9.7)	57 (14.1)	16 (14.8)	8 (5.9)
Other	152 (20.3)	21 (20.4)	48 (11.9)	20 (18.6)	63 (46.3)
First Language					
English	180 (23.9)	84 (81.6)	44 (10.9)	10 (9.3)	42 (30.9)
Other	572 (76.1)	19 (18.5)	360 (89.1)	98 (90.7)	94 (69.1)
Primary Language Spoken at Home					
English	175 (23.3)	84 (81.6)	48 (11.9)	12 (11.1)	31 (22.8)
Other	577 (76.7)	19 (18.5)	356 (88.1)	96 (88.9)	105 (77.2)
PHQ *M* (*SD*) Cronbach’s Alpha	7.90 (5.48).89	8.06 (6.24).93	7.57 (5.34).88	8.55 (5.80).89	8.20 (4.97).87
ATQ *M* (*SD*)Cronbach’s Alpha	68.34 (29.02).98	70.58 (33.27).99	66.78 (28.00).98	68.16 (30.51).98	71.25 (27.31).98
DAS *M* (*SD*)Cronbach’s Alpha	93.33 (17.01).84	97.67 (18.41).87	93.23 (16.81).84	94.16 (16.85).80	89.68 (16.00).84
ERQ-Suppression *M* (*SD*)Cronbach’s Alpha	17.47 (4.68).76	17.18 (4.83).82	17.00 (4.88).77	18.17 (4.56).71	18.48 (3.78).67
ERQ-Reappraisal *M* (*SD*)Cronbach’s Alpha	18.81 (4.14).81	18.51 (4.54).90	18.65 (4.22).81	19.29 (3.83).71	19.14 (3.81).77

*PHQ-8* Patient Health Questionnaire-8 total score, *ATQ-N* Automatic Thought Questionnaire-Negative total scores, *DAS* Dysfunctional Attitude Scale total score, *ERQ* Emotion Regulation Questionnaire, *ERQ – Reappraisal* ERQ Reappraisal totals score, *ERQ* Suppression total score

#### Patient health questionnaire-8 (PHQ; [46])

The PHQ-8 is an 8-item measure derived from the Diagnostic Statistical Manual IV (DSM-IV) criteria of depression to assess depressive symptoms. Participants rated the frequency of experiencing depressive symptoms over the last 2 weeks ranging from 0 (*not at all*) to 3 (*nearly every day*). Higher scores indicate greater severity of depressive symptoms. The excellent psychometric properties of PHQ-8 have been confirmed in previous studies [46]. In the current study, the PHQ-8 exhibited a Cronbach’s alpha of α = .89 for the entire sample. The PHQ-8 had good internal consistency within samples from each of the four continents, with alpha coefficients ranging from .87 to .93.

#### Dysfunctional attitude scale (DAS; [21])

The 24-item DAS assesses dysfunctional beliefs and assumptions implicated in depressive symptomology [47]. Participants rated their agreement (1 = *totally disagree;* 7 = *totally agree*), with several (“if…then”) statements (e.g., “I’m nothing if a person I love doesn’t love me”). After reversing negatively worded items, higher scores are reflective of greater negative attitudes. The DAS has been shown to be highly reliable and valid (e.g., [21, 48]). In the current study, the DAS had a Cronbach’s alpha internal reliability of α = .84 for the entire sample. Internal reliability ranged from .80 to .87 within samples among the four continents.

#### Emotion regulation questionnaire (ERQ; [31])

The ERQ is a 10-item scale designed to determine how people vary in their emotion regulation, especially in their usage of two emotion management techniques: cognitive reappraisal and emotional suppression. Participants indicated their agreement with different statements (e.g., “I keep my emotions to myself”; “When I’m faced with a stressful situation, I make myself think about it in a way that helps me calm”) on a scale from 1 (*strongly disagree*) to 7 (*strongly agree*). The ERQ has been frequently used in research among both general and clinical populations and has been shown to have excellent psychometric properties [31]. In the current study, the cognitive reappraisal and emotional suppression subscales demonstrated adequate to good internal consistency for the entire sample, with alpha coefficients of .81 and .76, respectively. The cognitive reappraisal subscale exhibited relatively high Cronbach’s alpha within samples from each of the four continents, ranging from .72 to .90. The emotional suppression subscale possessed acceptable internal consistency within samples from each of the four continents, with alpha coefficients ranging from .67 to .82.

#### Automatic thoughts questionnaire (ATQ-N; [49])

The ATQ-N is a 30-item instrument designed to assess how often people had negative thoughts in the past week. Participants expressed how much they agreed with various statements assessing automatic negative thoughts on a scale ranging from 1 (*not at all*) to 5 (*all of the time*) (e.g., “My future is bleak”; “I’m no good”). Higher scores correspond to greater frequency of negative thoughts. The ATQ-N has been used with both clinical and general populations, and has been shown to be both reliable and valid [6]. In the current study, the ATQ-N had Cronbach’s alpha internal reliability of α = .98 for the entire sample. Internal reliability within samples from each of the four continents ranged from .98 to .99.

### Data analysis

Person-mean imputation was used to impute missing values for each respondent on a given scale. The average of each respondent’s completed items was used to substitute the missing values within each scale. As noted above, 106 participants (12.35%) were excluded from the study. Participants who were excluded (*M* = 29.71, *SD* = 8.62) and those who were not (*M* = 31.52*, SD* = 9.07) did not significantly differ in age, *t*(855) = 1.94, *p* > .05. The two groups also did not contain different proportions of men and women, χ^2^(2, *N* = 858) = 1.41, *p* > .05. However, significant differences were observed between included and excluded participants on education, χ^2^(10, *N* = 858) = 19.26, *p* = .037; marital status, χ^2^(4, *N* = 858) = 12.18, *p* = .02; and income, χ^2^(6, *N* = 858) = 24.01 *p* < .001. Such analyses revealed that participants who were retained in the study were more likely to be educated and single and have higher income.

Given these statistically significant differences between included and excluded participants, correlation analyses were conducted to assess if education, marital status, and income were at all related to key variables of interest (i.e., PHQ, ATQ, DAS, cognitive reappraisal, and emotional suppression). Results indicated a significant relationship between education and PHQ scores, *r*(750) = −.08, *p* < .03. There were no other significant associates between education and other variables of interest (all *p*s > .05). Results also indicated a significant relationship between income and PHQ scores, *r*(750) = −.08, *p* < .04; no other associations between income and measures of interest were observed. No significant associations were observed between marital status and variables of interest (all *p*s > .05).

A necessary requirement in assessing continental differences is to demonstrate measurement invariance [50]. If a measure is variant across continents, differences could potentially be attributable to psychometric artifacts (e.g., scale understanding), rather than actual cross-national psychological differences. To assess invariance, we used the alignment method using Bayesian estimation [51, 52] using mPlus 8 [53]. Traditional multi-group CFAs often place the unnecessary and strict assumption of strict invariance, and in turn, an excessive number of modification indices are required to obtain acceptable fit statistics. Multi-group CFAs require strict invariance because configural invariance does not permit the comparison of factor means or factor variances. To address this concern, Asparouhov and Muthén [51] devised the alignment method, which estimates factor means and variances while using the measurement restrictions typically associated with configural invariance. The alignment method greatly simplifies invariance analyses by examining the extent that each item is invariant across each group. As a result, the alignment method is a superior alternative to multi-group CFA methods for examining measurement invariance when there are many groups, such as examining scores across country or continent [51]. We used alignment analysis to identify and remove non-invariant items from each of our scales. Theoretical justification for the removal of items is included in the discussion section.

## Results

### Uncorrected associations of depression symptoms, negative cognitions, and emotion regulation

Pearson correlations were used to examine associations of uncorrected scores on the PHQ-8 (depressive symptoms), ATQ-N (negative automatic thoughts), DAS (Dysfunctional attitudes), and ERQ-Suppression and ERQ-Reappraisal (use of expressive suppression and cognitive reappraisal strategies) in each of the four continents (Table 3). Depressive symptoms were positively associated with negative automatic thoughts and dysfunctional attitudes in people living on each of the four continents. Further, depressive symptoms were positively associated with the use of expressive suppression among participants on all continents.

**Table 3:** Uncorrected correction matrix

	North America (*n* = 103)				Europe (*n* = 404)				South America (*n* = 108)				Asia (*n* = 136)			
	1	2	3	4	1	2	3	4	1	2	3	4	1	2	3	4
1. PHQ-8	–				–				–				–			
2. ATQ-N	.79**	–			.75**	–			.77**	–			.68**	–		
3. DAS	.25*	.28**	–		.35**	.33**	–		.36**	.42**	–		.29**	.27**	–	
4. ERQ-Suppress	.34**	.39**	.20*	–	.22**	.22**	.18**	–	.42**	.31**	.21*	–	.28**	.13	.02	–
5. ERQ-Reappraise	.06	.08	.01	.47**	.07	−.13**	.01	.21**	.11	.04	.05	.25**	.06	−.01	.01	.34**

*PHQ-8* Patient Health Questionnaire-8 total score, *ATQ-N* Automatic Thought Questionnaire-Negative total scores, *DAS* Dysfunctional Attitude Scale total score, *ERQ* Emotion Regulation Questionnaire, *ERQ – Reappraisal* ERQ Reappraisal totals score, *ERQ* Suppression total score

* *p* < .05; ** *p* < .01

### Item reduction based on the alignment method

The PHQ alignment analyses indicated two items were subject to variant factor loadings across continents: “Feeling down, depressed, or hopeless” (item 2); and “Moving or speaking so slowly that other people could have noticed. Or the opposite; being so fidgety or restless that you have been moving around more than usual” (item 8).

The DAS alignment analyses showed that items 3 (“I should be happy all the time”) and 12 (“A person should be able to control what happens to him”) exhibited variant intercepts. Items 15 (“It is possible for a person to be scolded and not get upset”), 20 (“I do not need the approval of other people in order to be happy”), and 23 of the DAS (“A person doesn’t need to be well liked in order to be happy”) exhibited variant factor loadings.

The ATQ alignment analyses suggested that item 7 (“I wish I were a better person”) exhibited a variant intercept.

All items of the ERQ-Suppress and ERQ-Reappraise subscales exhibited invariance across continents, and therefore all items on these subscales were retained.

### Corrected associations of depression symptoms, negative cognitions, and emotion regulation

Correlations among measures of interest with variant items removed (i.e., corrected associations) are presented in Table 4. Depressive symptoms were positively correlated with negative automatic thoughts, dysfunctional attitudes, and emotional suppression across all four continents. Use of emotional suppression demonstrated a positive and significant relationship with ATQ scores in North American, European, and South American, but not among Asian participants. Use of emotional suppression was also positively and significantly related to DAS scores in European and South American, but not among North American and Asian participants. Apart from a small, negative correlation between use of cognitive reappraisal and negative automatic thoughts among European participants, reappraisal did not significantly correlate with any depression related constructs. Use of suppression and cognitive reappraisal was positively associated across continents.

**Table 4:** Correlation matrix with bias-corrected scores

	North America (*n* = 103)				Europe (*n* = 404)				South America (*n* = 108)				Asia (*n* = 136)			
	1	2	3	4	1	2	3	4	1	2	3	4	1	2	3	4
1. PHQ-8	–				–				–				–			
2. ATQ-N	.77**	–			.73**	–			.77**	–			.65**	–		
3. DAS	.25*	.26**	–		.36**	.34**	–		.31**	.44**	–		.27**	.29**	–	
4. ERQ-Suppress	.31**	.39**	.19	–	.21**	.22**	.20**	–	.43**	.30**	.21*	–	.30**	.13	.04	–
5. ERQ-Reappraise	.07	.07	.01	.47**	−.08	−.13**	.01	.21**	.12	.04	.06	.25**	.08	−.02	.03	.34**

*PHQ-8* Patient Health Questionnaire-8 total score, *ATQ-N* Automatic Thought Questionnaire-Negative total scores, *DAS* Dysfunctional Attitude Scale total score, *ERQ* Emotion Regulation Questionnaire, *ERQ – Reappraisal* ERQ Reappraisal totals score, *ERQ* Suppression total score

* *p* < .05; ** *p* < .01

### Comparative strength of associations of depression with negative cognitions and emotional regulation

Fisher’s *r*-to-*z* transformations were used to compare the strength of associations between depressive symptoms and corrected scores on the ATQ-N, DAS, and ERQ-Suppression among each of the four continents. The association between depressive symptoms and negative automatic thoughts was larger among North Americans than among Asians, *z* = 1.84, *p* = .03. The association between depressive symptoms and negative automatic thoughts was larger among South Americans than among Asians, *z* = 1.88, *p* = .03. No other significant differences were observed in the strength of associations between depressive symptoms and ATQ-N scores among other continents (all *p*s > .05).

No significant differences were observed in the strength of associations between depressive symptoms and DAS scores among the continents (all *p*s > .05).

The association between use of suppression and use of cognitive reappraisal was significantly stronger among North Americans than among South Americans, *z* = 1.82, *p* = .034, and stronger among North Americans than among Europeans, *z* = 2.66, *p* < .01. No other significant differences were observed (all *p*s > .05).

## Discussion

In the present study, we examined the relationships of depression symptoms with negative thoughts and use of two emotion regulation strategies among people residing in several countries on four different continents. Although the cognitive model of depression has been forwarded as a universal model of the experience of depression, very few studies to date have examined these foundational hypotheses outside of a Western context [7, 54]. This study is first to examine central hypotheses of the cognitive model of depression across such a wide range of regions around the world. Taken together, the results provide evidence that the key predictions of the cognitive model are applicable cross-continentally.

Depressive symptoms were positively correlated with negative automatic thoughts about self among each of the four continents examined. This result is consistent with Beck’s original cognitive model of depression [4], and provides strong support for this foundational hypothesis cross-continentally. Earlier research has found that self-reproach and guilt are atypical features of depression outside of Western contexts. This pattern of results has led some to conclude that the negative automatic thoughts that often accompany affective disorders in the West are rare in non-Judeo-Christian sufferers of depression [15, 16]. In a similar vein, Yap [55] has suggested that the absence of depressive guilt seen in Chinese people may be due to the projection of their guilt and feelings of worthlessness onto supernatural figures. Our results are inconsistent with these alternative explanations, but are supportive of the cognitive model.

Some researchers have suggested that cross-national findings inconsistent with the cognitive model suffer from key methodological limitations, which may in turn mask associations between depressive symptoms and negative self-thoughts [56, 57]. One hypothesis is that outside of the West, there is little vernacular language that effectively captures self-referent automatic negative thoughts. Consistent with this hypothesis, Matthey et al. [58] found that participants of Arabic descent expressed psychological symptoms of depression more readily when using self-report questionnaires (compared to face-to-face interviews with researchers or clinicians). It may be the case that negative self-referent cognitions are central to the experience of depression cross-nationally, but may not be accurately presented, endorsed, disclosed, or assessed due to local differences in language, or cultural rules regarding self- disclosure. Our results provide some evidence to support this notion.

Depressive symptoms were positively associated with dysfunctional attitudes across continents, and the degree of this association was relatively smaller in comparison to the association of depressive symptoms and negative automatic thoughts. This finding is consistent with Beck’s model of depression [8, 10], which states that negative automatic thoughts are proximate to depression symptoms, and are therefore expected to be more strongly correlated with depression than dysfunctional attitudes. Our results are consistent with several cross-national studies demonstrating the relationship of depression symptoms with dysfunctional attitudes (e.g., [19, 59, 60]).

Use of cognitive reappraisal was not associated with depression symptoms among people of all four continents. However, previous research on the effects of cognitive reappraisal among people of Western descent suggest that use of this strategy is adaptive, and thus negatively associated with depression [37, 61]. It is possible the effectiveness of a particular emotion regulation strategy in ameliorating depressive symptoms depends on cultural norms as they interact with individual psychology or intrapsychic factors. For example, in Eastern cultures, the suppression of negative emotions is encouraged to maintain social harmony [38], and evidence suggests that expressive suppression is less detrimental in Eastern cultures than in Western cultures [39]. Therefore, cognitive reappraisal may be a less effective emotion regulation strategy among the former. Further evidence suggests that the relationship of cognitive reappraisal and depression symptoms may be moderated by stress [35]. Troy et al. [35] provide evidence suggesting that cognitive reappraisal is protective against depression only in times of high stress. In the present study, we did not examine stress as a moderator of the relationship between use of cognitive reappraisal and depression, although future cross-national research examining the protective effects of cognitive appraisal would be illuminating.

Depressive symptoms were positively associated with use of expressive suppression among people living on all continents. This suggests that expressive suppression is a maladaptive emotion regulation strategy regardless of peoples’ region of residence**.** This finding is consistent with research showing that the use of this strategy is often counterproductive for the management of depression [31, 62]. The use of suppression and reappraisal were correlated. It is possible that people who are more distressed (e.g., showing higher symptoms of depression) may be employing more emotion regulation strategies than others who are not endorsing heightened symptoms of the disorder.

A notable aspect of this study is the use of alignment analyses to assess and correct for invariance in items across continents. This analysis revealed that several items in the measured used were variant among the four different samples. For example, PHQ items 2 (depressed mood) and 8 (psychomotor abnormalities) were both found to be variant across continents, prompting us to remove them from the analyses. Corrections for invariance on items is of particular importance given inconsistent previous findings about the psychometric generalizability of PHQ measures cross-nationally. For example, some reseach [63] demonstes that item 8 on the PHQ (psychomotor abnormalities) is variant across different cultures. Others have shown no such invariance in the PHQ (e.g., [64].

One potential source of invariance among PHQ items is the nature of the items themselves. Some studies [65] suggest that responses on affective items of depression scales, such as item 2 (depressed mood) of the PHQ, are often fused with somatic items. Given that these concepts are not clearly distinguished, such items may be of particular psychometric concern when used cross-nationally. Our alignment analysis revealed that most positivity-valenced items of the DAS (e.g., “I do not need the approval of other people in order to be happy”) were variant across continents. This finding is consistent with the literature that suggests that reversed-coded items on psychological measures are weaker psychometrically [66, 67]. Taken together, the results of this and other studies suggest that invariance of items is of particular concern in depression research (and mental health research more generally) and requires further empirical attention.

The present study extends and replicates extant literature in several important ways. First, this study is the first relatively large scale, cross-continental study of foundational hypotheses related to the cognitive model of depression. Second, this study is one of the first to employ the alignment method in creating invariant measurement scores across several groups—an important consideration given previous inconsistencies in depression measures used cross-nationally. Third, we used a continental-control sample (i.e., a North American sample) to compare responses from respondents living on continents that are not very well researched relative to Western people (i.e., South America; Asia). Finally, we used several commonly used and well-validated measures of the constructs of interest, making possible comparisons with earlier work using identical scales.

Despite the results of the present study, there are several limitations that require noting. First, participants were recruited through an online, crowdsourcing website, necessitating that people have access to a computer and internet. This recruitment approach limits the generalizability of our findings. Second, we administered all scales in English. Therefore, participants had to have an eighth-grade or equivalent English reading and comprehension level to be able to respond appropriately to the items. These language requirements are potentially problematic in several ways (e.g., generalizability to the majority, non-English speaking participants living on these continents; assumption that most people understood the items). Participants’ responses might have been different if the measures were administered in their native language. This could have impacted the accuracy of our findings. Indeed, the sample was somewhat homogenous in its composition (e.g., mostly White and male). Third, we did not assess people living in Africa or Australia. Fourth, there is vast heterogeneity in cultural, social, and political factors that govern the behavior and cognition of people within each continent, further limiting generalizability, especially within regions. However, this heterogeneity is a key strength of the study: that such diverse populations across the world showed similar evidence in support of the cognitive model is compelling. Fifth, it is not clear whether participants residing in various continents were internalized the majority culture of their respective continent. Even though this study utilized a relatively large sample, the majority of participants were from Europe. This further limit the generalizability of the findings. Sixth, we did not examine the associations of negative cognitions, emotional regulation, and depression symptoms across specific cultures to identify the potential impact of individualism and collectivism. Cognitive correlates of depression may demonstrate different associations with depression symptoms among members of a specific culture within a particular continent. Seventh, we examined the association of ethnicity with depressive symptoms, negative automatic thoughts, dysfunctional attitudes, and use of cognitive reappraisal and expressive suppression (see Additional file 1: Ancillary Analyses); however, we did not examine the potential impact of ethnicity on the association of depressive symptoms with negative cognitions and use of two emotion regulation strategies. Finally, we examined the association of age with our study variables and its potential impact on the relationships of depression symptoms with negative thoughts and use of two emotion regulation strategies (see Ancillary Analyses). However, we did not include religion in our analyses, which may have affected the strength of the observed associations.

Our results provide some directions for future research. It would be useful to replicate the findings reported herein with people living in specific countries or affected by specific cultural climates (e.g., comparing ethnic minorities and ethnic majorities within countries). Future studies could also examine the underlying assumptions of CBT across individualistic and collectivistic cultures to identify whether such assumptions hold across specific cultural milieus. Further, future work could examine other foundational hypotheses of the cognitive model. For instance, we did not examine any aspects of the selective processing hypothesis, which states that people demonstrating elevated depression scores would also demonstrate skewed processing of information in attention, retention, and recall [8]. Moreover, we could not assess the causal role of cognition in the onset and maintenance of depression symptoms. Thus, future studies employing longitudinal designs would benefit from examination of such hypotheses across cultures and regions. We did not examine the potential impact of religion and ethnicity on the relationships of depression symptoms with negative thoughts and use of two emotion regulation strategies. Therefore, future studies should examine the potential impact of these variables on the strength of such associations. Finally, future studies should examine cognitive correlates of depression (e.g., dysfunctional attitudes) across various ethnic groups to identify which aspect of these cognitions may be different, and how that would impact individual levels of symptoms.

## Conclusions

While the results of the present study may offer preliminary cross-national support for foundational hypotheses of the cognitive model of depression, the use of country as a proxy for culture is common despite its questionable nature [68]. Scholars have argued that country is not an appropriate proxy for culture, as there are greater intra-country cultural variations than there are inter-country variations [69]. Therefore, the use of country as a proxy of culture may put researchers at “the risk of not capturing all of the relevant cultural factors that might lend support to (or that might discredit) their theories and hypotheses” ([70], p. 176). Taras et al. [69] found that cultural dimensions demonstrate stronger relationships with demographics and environmental factors than with country. In particular, they suggest that factors, such as socioeconomic status, globalization, and economic freedom, to name a few, are better clustering dimensions of culture than country [69]. Despite this, it is not clear how such dimensions can be used to measure culture. Even though researchers are offering new insights on the measurement of culture, a general agreement on the best ways of measuring it is still lacking [68]. Beugelsdijk, Kostova, and Roth [71] have mentioned that “to abandon the country as the unit of analysis may be too far-fetched” as suggested by other researchers (p. 33).

The present study provides evidence indicating that the foundational hypotheses of Beck’s cognitive model of depression are consistent among people living around the world. This evidence has important implications to theory and practice of cognitive therapy across different regions. Given the close link between depression and negative automatic thoughts about self, practitioners of CBT practicing on the four examined continents may benefit from consistently employing techniques that help clients identify and challenge their patients’ negative thoughts. Of course, larger scale, more specific, and more demographically representative trials are needed to replicate findings of the present study, but the findings are certainly promising for the generalizability of such treatments as cognitive behavioral therapy. The present study did not actually test cross-cultural effectiveness of CBT, but rather its core assumptions, which remains an important area of investigation.

## Supplementary information

**Additional file 1.** Ancillary Analyses. These analyses provide information on how age and ethnicity were associated with the study variables. Further, these analyses indicate the differences in the association of depressive symptoms with negative cognitions of depression and use of emotion regulation strategies among Eastern and Western European countries.

## Data Availability

Data are available to download from the following link: https://cutt.ly/SrqRmqX. The datasets used and/or analyzed during the current study are also available from the corresponding author on reasonable request.

## Abbreviations

ATQ-N: Negative Automatic Thought Questionnaire
CBT: Cognitive-Behavioural Therapy
DAS: Dysfunctional Attitude Scale
DSM-5: Diagnostic Statistical Manual 5th edition
DSM-IV: Diagnostic Statistical Manual IV
ERQ: Emotion Regulation Questionnaire
PHQ: Patient Health Questionnaire

## References

[1] Kessler RC, Bromet EJ (2013). The epidemiology of depression across cultures. Annu Rev Public Health.

[2] Friedrich M (2017). Depression Is the Leading Cause of Disability Around the World. JAMA.

[3] Cuijpers P, Berking M, Andersson G, Quigley L, Kleiboer A, Dobson KS (2013). A meta-analysis of cognitive-behavioural therapy for adult depression, alone and in comparison with other treatments. Can J Psychiatry.

[4] Beck AT. Cognitive therapy of depression. New York: Guilford Press; 1979. 10.1046/j.1440-1614.2002.t01-5-01015.x 11982561

[5] Beshai S, Wallace LM, Mcdougall KH, Waldmann K, Stea JN (2016). Reduced contact cognitive-behavioral interventions for adult depression: a review. J Psychol.

[6] Beshai S, Dobson KS, Adel A (2012). Cognition and dysphoria in Egypt and Canada: an examination of the cognitive triad. Can J Behav Sci.

[7] Beshai S, Dobson KS, Adel A, Hanna N (2016). A cross-cultural study of the cognitive model of depression: cognitive experiences converge between Egypt and Canada. PLoS One.

[8] Clark DA, Beck AT. Scientific foundations of cognitive theory and therapy of depression. New York: Wiley; 1999.

[9] American Psychiatric Association. Diagnostic and statistical manual of mental disorders (5th ed.). Arlington: American Psychiatric Association; 2013.

[10] Beck Aaron T. (2008). The Evolution of the Cognitive Model of Depression and Its Neurobiological Correlates. American Journal of Psychiatry.

[11] Beck AT. The development of depression: a cognitive model. In: Friedman RJ, Katz MM (Eds.). The psychology of depression: contemporary theory and research. Washington, DC: Winston-Wiley; 1974. p. 3–27.

[12] Dobson Keith S., Shaw Brian F. (1987). Specificity and stability of self-referent encoding in clinical depression. Journal of Abnormal Psychology.

[13] Kendall Philip C., Howard Bonnie L., Hays Rebecca C. (1989). Self-referent speech and psychopathology: The balance of positive and negative thinking. Cognitive Therapy and Research.

[14] Haaga David A., Dyck Murray J., Ernst Donald (1991). Empirical status of cognitive theory of depression. Psychological Bulletin.

[15] ​Sami M, El-Gawad A. Transcultural psychiatry in Egypt. In: Al-Issa I (Ed.), Handbook of culture and mental illness: An international perspective. Madison: International Universities Press, 1995. p. 56–64.

[16] Stompe T., Ortwein-Swoboda G., Chaudhry H.R., Friedmann A., Wenzel T., Schanda H. (2001). Guilt and Depression: A Cross-Cultural Comparative Study. Psychopathology.

[17] Wang T, Wang N, Hu H, Feng Z, Liu Y. Relationship of depression, automatic thoughts and personality in medical college students. J Third Med Univ. 2007;5:442–4.

[18] Pan Jia-Yan, Ye Shengquan, Ng Petrus (2015). Validation of the Automatic Thoughts Questionnaire (ATQ) Among Mainland Chinese Students in Hong Kong. Journal of Clinical Psychology.

[19] Şahin Nesrin H., Şahin Nail (1992). Reliability and validity of the Turkish version of the automatic thoughts questionnaire. Journal of Clinical Psychology.

[20] TANAKA NAO, UJI MASAYO, HIRAMURA HIDETOSHI, CHEN ZI, SHIKAI NORIKO, KITAMURA TOSHINORI (2006). Cognitive patterns and depression: Study of a Japanese university student population. Psychiatry and Clinical Neurosciences.

[21] Weissman AN, Beck AT. Development and validation of the dysfunctional attitude scale: a preliminary investigation. Chicago: Paper presented at the meeting of the Association for the Advancement of Behavior Therapy; 1978.

[22] Beshai Shadi, Prentice Jennifer L., Swan Jennifer L., Dobson Keith S. (2014). The Effects of Dysphoria and Personality on Negative Self-Referent Attitudes and Perceptions of the Attitudes of Others. The Journal of Psychology.

[23] Goldberg Joseph F., Gerstein Rachel K., Wenze Susan J., Welker Tara M., Beck Aaron T. (2008). Dysfunctional Attitudes and Cognitive Schemas in Bipolar Manic and Unipolar Depressed Outpatients. The Journal of Nervous and Mental Disease.

[24] Dobson Keith S., Shaw Brian F. (1986). Cognitive assessment with major depressive disorders. Cognitive Therapy and Research.

[25] Mukhtar Firdaus, Oei Tian P.S. (2010). Exploratory and confirmatory factor validation of the Dysfunctional Attitude Scale for Malays (DAS-Malay) in Malaysia. Asian Journal of Psychiatry.

[26] Kallay E, Dégi CL, Vincze AE. Dysfunctional attitudes, depression and quality of life in a sample of Romanian. J Cogn Behav Psychother. 2007;7(1):95–106.

[27] Cuéllar MP, Guerrero MNV, Marfil MNP, Uclés IR. Cronicidad de los trastornos del estado de ánimo: relaciones con actitudes cognitivas disfuncionales y con alteraciones de la personalidad Chronicity of mood disorders: their. Clínica y salud: Revista de psicología clínica y salud. 2007;18(2):203–19.

[28] Fatemi Azadeh S., Younesi Seyed Jalal, Azkhosh Manouchehr, Askari Ali (2010). Comparison of dysfunctional attitudes and social adjustment among infertile employed and unemployed women in Iran. International Journal of Psychology.

[29] Thomas Justin, Altareb Belkeis (2011). Cognitive vulnerability to depression: An exploration of dysfunctional attitudes and ruminative response styles in the United Arab Emirates. Psychology and Psychotherapy: Theory, Research and Practice.

[30] Dobson KS. Handbook of cognitive-behavioral therapies. New York: Guilford Press; 2009.

[31] Gross James J., John Oliver P. (2003). Individual differences in two emotion regulation processes: Implications for affect, relationships, and well-being. Journal of Personality and Social Psychology.

[32] Gross James J. (1998). Antecedent- and response-focused emotion regulation: Divergent consequences for experience, expression, and physiology. Journal of Personality and Social Psychology.

[33] ​Gross JJ. Handbook of emotion regulation. New York: Guilford publications; 2013.

[34] ​Gross JJ, Thompson RA. Emotion regulation: Conceptual foundations. In: Gross JJ (Ed.). Handbook of emotion regulation. New York: The Guilford Press; 2007. p. 3–24.

[35] Troy Allison S., Wilhelm Frank H., Shallcross Amanda J., Mauss Iris B. (2010). Seeing the silver lining: Cognitive reappraisal ability moderates the relationship between stress and depressive symptoms. Emotion.

[36] Haga Silje Marie, Kraft Pål, Corby Emma-Kate (2007). Emotion Regulation: Antecedents and Well-Being Outcomes of Cognitive Reappraisal and Expressive Suppression in Cross-Cultural Samples. Journal of Happiness Studies.

[37] Moore Sally A., Zoellner Lori A., Mollenholt Niklas (2008). Are expressive suppression and cognitive reappraisal associated with stress-related symptoms?. Behaviour Research and Therapy.

[38] Soto José A., Perez Christopher R., Kim Young-Hoon, Lee Elizabeth A., Minnick Mark R. (2011). Is expressive suppression always associated with poorer psychological functioning? A cross-cultural comparison between European Americans and Hong Kong Chinese. Emotion.

[39] Butler Emily A., Lee Tiane L., Gross James J. (2007). Emotion regulation and culture: Are the social consequences of emotion suppression culture-specific?. Emotion.

[40] Kwon Hoin, Yoon K. Lira, Joormann Jutta, Kwon Jung-Hye (2013). Cultural and gender differences in emotion regulation: Relation to depression. Cognition & Emotion.

[41] Marsh Herbert W., Guo Jiesi, Parker Philip D., Nagengast Benjamin, Asparouhov Tihomir, Muthén Bengt, Dicke Theresa (2018). What to do when scalar invariance fails: The extended alignment method for multi-group factor analysis comparison of latent means across many groups. Psychological Methods.

[42] Chandler Jesse, Shapiro Danielle (2016). Conducting Clinical Research Using Crowdsourced Convenience Samples. Annual Review of Clinical Psychology.

[43] Le J, Edmonds A, Hester V, Biewald L. Ensuring quality in crowdsourced search relevance evaluation: the effects of training question distribution. Paper presented at the SIGIR 2010 workshop on crowdsourcing for search evaluation; 2010.

[44] Mishra Sandeep, Carleton R. Nicholas (2017). Use of online crowdsourcing platforms for gambling research. International Gambling Studies.

[45] de Winter J.C.F., Kyriakidis M., Dodou D., Happee R. (2015). Using CrowdFlower to Study the Relationship between Self-reported Violations and Traffic Accidents. Procedia Manufacturing.

[46] Kroenke Kurt, Strine Tara W., Spitzer Robert L., Williams Janet B.W., Berry Joyce T., Mokdad Ali H. (2009). The PHQ-8 as a measure of current depression in the general population. Journal of Affective Disorders.

[47] Power M.J., Katz R., McGuffin P., Duggan C.F., Lam D., Beck A.T. (1994). The Dysfunctional Attitude Scale (DAS). Journal of Research in Personality.

[48] Beshai Shadi, Branco Laura D., Dobson Keith S. (2013). Lemons Into Lemonade: Development and Validation of an Inventory to Assess Dispositional Thriving. Europe’s Journal of Psychology.

[49] Hollon Steven D., Kendall Philip C. (1980). Cognitive self-statements in depression: Development of an automatic thoughts questionnaire. Cognitive Therapy and Research.

[50] Vandenberg Robert J., Lance Charles E. (2000). A Review and Synthesis of the Measurement Invariance Literature: Suggestions, Practices, and Recommendations for Organizational Research. Organizational Research Methods.

[51] Asparouhov Tihomir, Muthén Bengt (2014). Multiple-Group Factor Analysis Alignment. Structural Equation Modeling: A Multidisciplinary Journal.

[52] ​Muthén B, Asparouhov T. IRT studies of many groups: The alignment method. Front Psychol. 2014;5:1–7. 10.3389/fpsyg.2014.00978 PMC4162377 25309470

[53] ​Muthén LK, Muthén B. mPlus 8.00 [computer software]. Los Angeles; 2017.

[54] Beshai Shadi, Clark Cameron M., Dobson Keith S. (2012). Conceptual and Pragmatic Considerations in the Use of Cognitive-Behavioral Therapy with Muslim Clients. Cognitive Therapy and Research.

[55] Yap PM. Phenomenology of affective disorder in chinese and other cultures. In: deReuck AVS, Porter R (Eds.). Transcultural Psychiatry. London, England: J. & A. Churchill. 1965;84–108.

[56] Hamdi Emad, Amin Yousreya, Abou-Saleh Mohammed T (1997). Problems in validating endogenous depression in the Arab culture by contemporary diagnostic criteria. Journal of Affective Disorders.

[57] Sulaiman Sultana O. Y., Bhugra Dinesh, de Silva Padmal (2001). Perceptions of Depression in a Community Sample in Dubai. Transcultural Psychiatry.

[58] Matthey Stephen, Barnett Bryanne E. W., Elliott Amanda (1997). Vietnamese and Arabic Women's Responses to the Diagnostic Interview Schedule (Depression) and Self-Report Questionnaires: Cause for Concern. Australian & New Zealand Journal of Psychiatry.

[59] Ohrt Torbjörn, Thorell Lars-Håkan (1998). Dysfunctional Attitude Scale (DAS). Psychometrics and Norms of the Swedish Version. Scandinavian Journal of Behaviour Therapy.

[60] ​Liu Y-L. The role of perceived social support and dysfunctional attitudes in predicting Taiwanese adolescents' depressive tendency. Adolescence. 2002;37(148):823–34. 12564832

[61] Garnefski Nadia, Kraaij Vivian (2006). Relationships between cognitive emotion regulation strategies and depressive symptoms: A comparative study of five specific samples. Personality and Individual Differences.

[62] Ehring Thomas, Tuschen-Caffier Brunna, Schnülle Jewgenija, Fischer Silke, Gross James J. (2010). Emotion regulation and vulnerability to depression: Spontaneous versus instructed use of emotion suppression and reappraisal. Emotion.

[63] Huang Frederick Y., Chung Henry, Kroenke Kurt, Delucchi Kevin L., Spitzer Robert L. (2006). Using the patient health questionnaire-9 to measure depression among racially and ethnically diverse primary care patients. Journal of General Internal Medicine.

[64] Baas Kim D., Cramer Angélique O.J., Koeter Maarten W.J., van de Lisdonk Eloy H., van Weert Henk C., Schene Aart H. (2011). Measurement invariance with respect to ethnicity of the Patient Health Questionnaire-9 (PHQ-9). Journal of Affective Disorders.

[65] Beshai Shadi, Dobson Keith S., Adel Ashraf (2013). Psychometric properties of the Center for Epidemiologic Studies Depression Scale in an Egyptian student sample. Middle East Current Psychiatry.

[66] Rodebaugh Thomas L., Woods Carol M., Heimberg Richard G. (2007). The Reverse of Social Anxiety Is Not Always the Opposite: The Reverse-Scored Items of the Social Interaction Anxiety Scale Do Not Belong. Behavior Therapy.

[67] Carlson Mike, Wilcox Rand, Chou Chih-Ping, Chang Megan, Yang Frances, Blanchard Jeanine, Marterella Abbey, Kuo Ann, Clark Florence (2011). Psychometric properties of reverse-scored items on the CES-D in a sample of ethnically diverse older adults. Psychological Assessment.

[68] Caprar Dan V, Devinney Timothy M, Kirkman Bradley L, Caligiuri Paula (2015). Conceptualizing and measuring culture in international business and management: From challenges to potential solutions. Journal of International Business Studies.

[69] Taras Vas, Steel Piers, Kirkman Bradley L. (2016). Does Country Equate with Culture? Beyond Geography in the Search for Cultural Boundaries. Management International Review.

[70] Schaffer Bryan S., Riordan Christine M. (2003). A Review of Cross-Cultural Methodologies for Organizational Research: A Best- Practices Approach. Organizational Research Methods.

[71] Beugelsdijk Sjoerd, Kostova Tatiana, Roth Kendall (2016). An overview of Hofstede-inspired country-level culture research in international business since 2006. Journal of International Business Studies.

