# Supplementary material for: Associations of negative cognitions, emotional regulation, and depression symptoms across four continents: International support for the cognitive model of depression
Source: BMC Psychiatry. 2020 Jan 13;20:18. doi: 10.1186/s12888-019-2423-x (PMC6958702; doi:10.1186/s12888-019-2423-x)
Supplement: Supplementary file 1 — Additional file 1. Ancillary Analyses. These analyses provide information on how age and ethnicity were associated with the study variables. Further, these analyses indicate the differences in the association of depressive symptoms with negative cognitions of depression and use of emotion regulation strategies among Eastern and Western European countries. [file 12888_2019_2423_MOESM1_ESM.docx]

**Additional File: Ancillary Analyses**

**The Associations between Age and Study Variables**

We conducted a correlational analysis to examine the association between age and the measures of interest. The results revealed a negative and significant association between age and depressive symptoms, *r*(749) = -.15, *p* < .001; age and negative automatic thoughts, *r*(749) = -.08, *p* < .03; and age and use of expressive suppression, *r*(749) = -.14, *p* < .001. Age and dysfunctional attitudes were positively associated, *r*(749) = .12, *p* < .01. However, the relationship between age and cognitive reappraisal was not statistically significant, *p* > .05.

A partial correlational analysis was conducted with age as a control variable to better identify the association of depressive symptoms with negative automatic thoughts, dysfunctional attitudes, and use of expressive suppression and cognitive reappraisal. The results indicated a positive and significant relationship with depressive symptoms and negative automatic thoughts *r*(748) = .75, *p* < .001; depressive symptoms and dysfunctional attitudes, *r*(748) = .31, *p* < .001; and depressive symptoms and use of expressive suppression, *r*(748) = .27, *p* < .001. No other significant associations were found (*p* > .05).

**Negative Cognitions and Emotion Regulation Strategies Among Eastern and Western European Countries**

We conducted several independent sample t-test to compare participants from Eastern and Western European countries on depressive symptoms, negative automatic thoughts, dysfunctional attitudes, and use of expressive suppression and cognitive reappraisal. The results revealed significant differences between participants from Eastern and Western European countries in negative automatic thoughts, *t*(383) = 2.10, *p* < .04. Participants from Western European countries had more negative automatic thoughts than those from Eastern European countries. There were no other significant differences between Eastern and Western European participants (all *p*s > .05).

**The Association between Ethnicity and Study Variables**

we conducted several correlational analyses to examine how ethnicity was associated with depressive symptoms, negative automatic thoughts, dysfunctional attitudes, and use of emotion regulation strategy of reappraisal and expressive suppression. There was a relatively low correlation between ethnicity and depressive symptoms, *r*(750) = .09, *p* < .02; ethnicity and cognitive reappraisal, , *r*(750) = .12, *p* < .01; and ethnicity and emotional suppression, *r*(750) = .12, *p* < .01. There were no other significant associations between ethnicity and other variables of interest (all *p*s > .05).
